# The Kerboull cross-plate: from the birth of a concept to the current indications

**DOI:** 10.1007/s00264-026-07059-9

**Published:** 2026-10-14

**Authors:** Julien Wegrzyn, Luc Kerboull, Jacques Béjui-Hugues, Moussa Hamadouche

**Affiliations:** 1 Department of Orthopedic Surgery and Traumatology, Lausanne University Hospital and University of Lausanne, Lausanne, Switzerland; 2 Clinique Arago, Paris, France; 3 Department of Orthopaedic and Reconstructive Surgery, Clinical Orthopaedics Research Center, Hôpitaux Universitaires Paris Centre (HUPC), Site Cochin-Port Royal, Assistance Publique-Hôpitaux de Paris (AP-HP), Université de Paris, Paris, France

**Keywords:** Acetabular reinforcement device, Kerboull cross-plate, Revision total hip arthroplasty, Bone allograft, Dual mobility cup

## Abstract

**Purpose:**

Reconstruction of major acetabular bone loss remains a demanding challenge during revision total hip arthroplasty. Since its original description, the Kerboull acetabular reinforcement device, also known as the Kerboull cross-plate or cage, has been developed to restore the native hip centre of rotation, to protect bone graft from early mechanical overload, and to allow cementation of a conventional full-polyethylene or a dual mobility cup.

**Methods:**

This narrative review traces the evolution of the Kerboull cross-plate from its original conception to its current indications.

**Results:**

We describe the biomechanical rationale of the device, supported by finite-element analyses demonstrating a marked load-sharing effect that protects the underlying bone graft in comparison with other reinforcement devices or unprotected cemented cups. We detail the surgical technique, as originally reported by Kerboull and Hamadouche, together with subsequent refinements aimed at standardising plate positioning. The principles governing graft selection are discussed: segmental structural defects require a structural graft, whereas morselised graft should be reserved for the filling of contained cavitary defects. The biological and mechanical quality of the graft is dependent on its processing, cryopreserved femoral head allograft being preferable to gamma-irradiated allograft. Finally, we review the mid- to long-term outcomes reported with the Kerboull cross-plate, including the more recent trend towards cementing a dual mobility cup within the device to reduce the risk of instability, and we outline the current indications of this technique of reconstruction.

**Conclusion:**

The Kerboull cross-plate retains its place as a reliable and time-tested option within the modern armamentarium for severe acetabular bone loss reconstruction.

## Introduction

Acetabular bone loss reconstruction is one of the most demanding challenges encountered during revision total hip arthroplasty (RTHA), and its clinical burden is expected to increase with the growing number of primary and revision procedures performed worldwide [1]. Acetabular bone defects are usually classified according to their location and severity, most commonly using the American Academy of Orthopaedic Surgeons (AAOS) system or the Paprosky classification, and the choice of reconstruction technique is largely dictated by the amount of remaining host bone able to provide primary mechanical support and osseointegration capacity for the acetabular construct used during revision [2, 3]. When a porous-coated cementless cup cannot achieve adequate host-bone coverage, several options have been developed, including jumbo cups, metal augments, cup-cage constructs, custom-made triflange components, and acetabular reinforcement devices combined with bone graft and a cemented cup [4, 5].

**Fig. 1:**
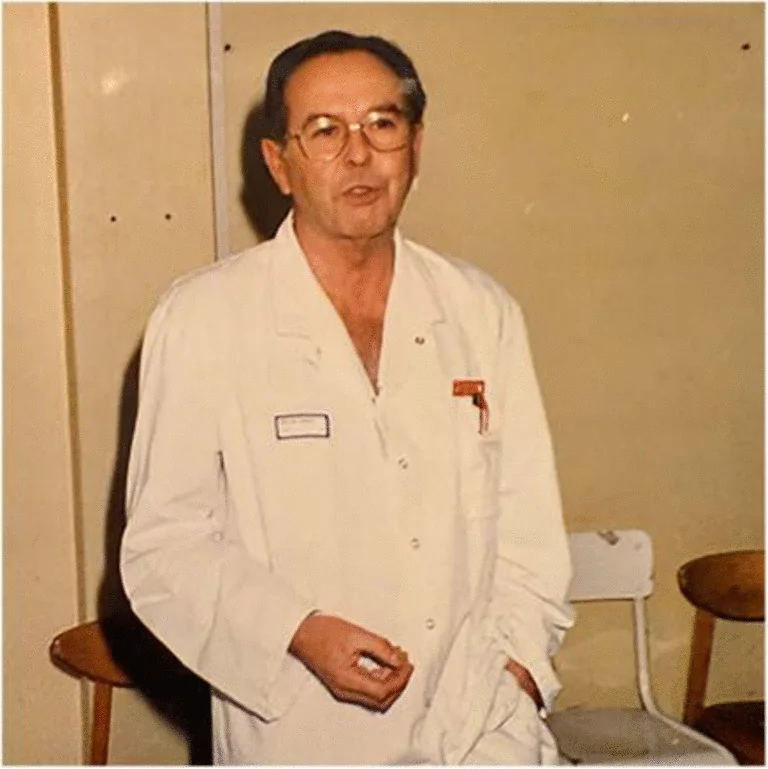
Professor Marcel Kerboull (1934–2020). Image used with permission from the Kerboull family [9]

Among the reinforcement devices, the Ganz ring, the Burch–Schneider antiprotrusio cage and the Kerboull acetabular reinforcement device, or Kerboull cross-plate or cage, remain the most widely used constructs, each combining an iliac fixation plate with an inferior hook or an ischial flange intended to achieve additional primary stability and increase fixation at the obturator foramen or the ischium. Since its introduction in Cochin University Hospital (Paris, France) in 1974 by Professor Marcel Kerboull (Fig. 1), the Kerboull cross-plate has accumulated one of the longest track records among acetabular reinforcement devices, and it has been the subject of a substantial body of literature describing its design rationale, biomechanical behaviour, and mid- to long-term clinical results, both in France and, notably, in Japan, where a modified Kerboull-type (KT) plate has been extensively used and studied [4, 5]. More recently, cementation of a dual mobility cup within the device has been proposed by Wegrzyn to further reduce the risk of postoperative dislocation, extending the indications of the construct to some of the most complex revision situations [6–8].

The aim of this narrative review is to summarise, for the practicing hip surgeon, the historical development of the Kerboull cross-plate, its biomechanical rationale, the surgical technique as originally described and subsequently refined, the principles that should govern the choice and quality of the bone graft used with the device, and the clinical outcomes that support its current indications.

## Birth of the concept: historical background and design

**Fig. 2:**
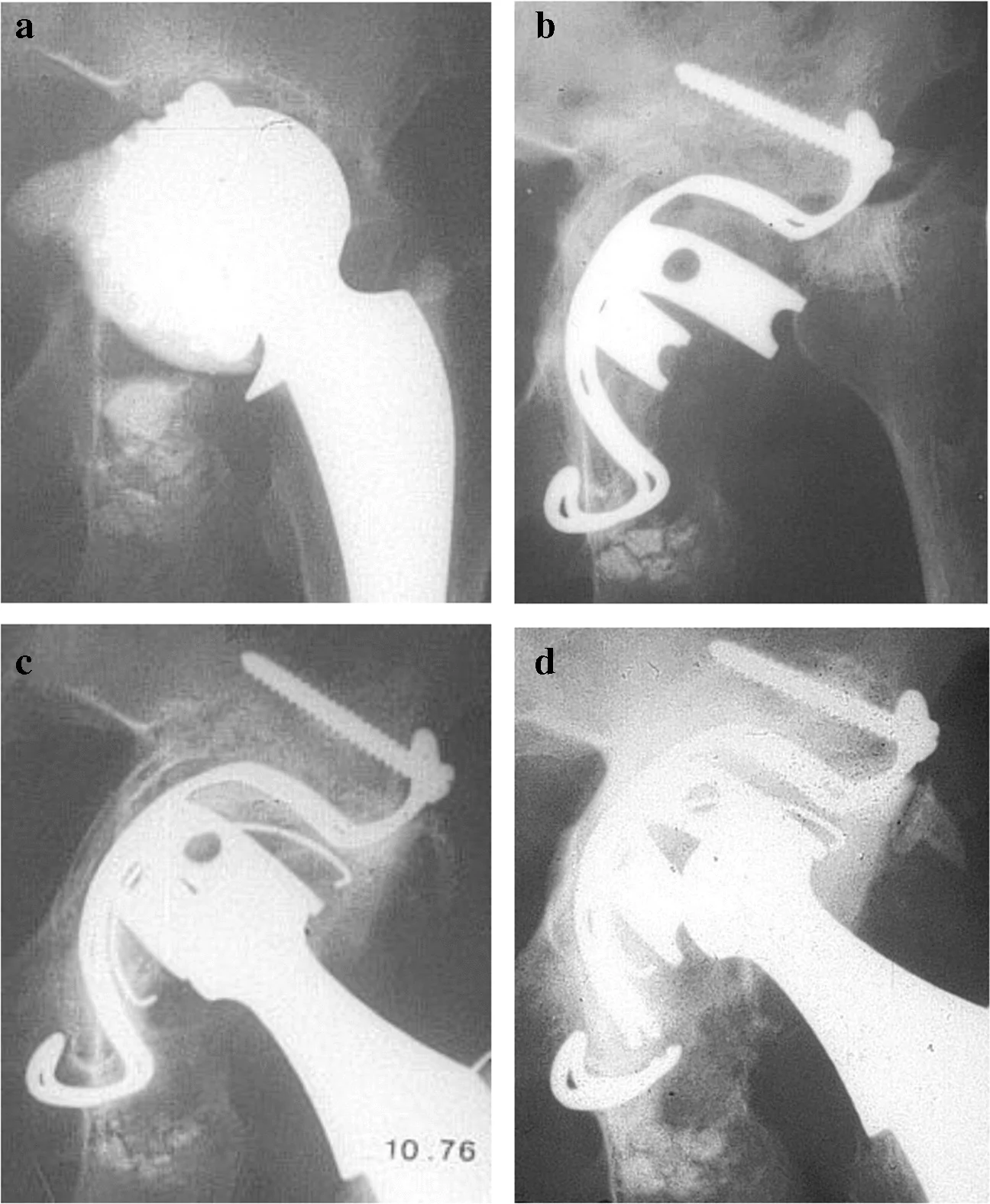
Historical case illustrating the first clinical use of the Kerboull acetabular reinforcement device. **A** Preoperative radiograph showing aseptic loosening of a metal-on-metal total hip arthroplasty associated with a transverse fracture of the acetabulum. **B** The first “hand-made” Kerboull cross-plate, used by Professor Marcel Kerboull in 1974 to treat this transverse acetabular fracture; the acetabulum was reconstructed with allograft and the plate, and a total hip arthroplasty was implanted six months later. **C** Result at 2-year follow-up showing consolidation of the transverse fracture and favourable incorporation of the allograft with a restored hip centre. **D** Result at 26-year follow-up showing polyethylene wear, partial graft resorption and limited superior migration of the construct with fracture of the hook; should another revision be required, the restored bone stock would allow a comparatively straightforward reconstruction

The Kerboull acetabular reinforcement device was developed by Professor Marcel Kerboull in 1974 [10], in the Department of Orthopedic Surgery of Cochin University Hospital in Paris – France, initially to address the reconstruction of major, combined segmental and cavitary acetabular defects encountered during RTHA (Fig. 2). The construct and its underlying surgical philosophy were formalised in the French surgical-technique literature by Kerboull and Kerboull [11], and were subsequently reported in the English-language literature in a comprehensive series of 60 consecutive revision THAs performed between 1980 and 1987, using bulk allograft bone supported by the device in 53 patients with AAOS type III (48 hips) or type IV (12 hips) defects [4]. In that landmark series, the reported survival rate free of aseptic loosening was approximately 92% at 13-year follow-up [4, 12].

The design of the Kerboull cross-plate includes a four-branched hemispherical construct manufactured from 316L stainless steel, resulting from the orthogonal crossing of two hemispherical plates: a vertical component, comprising a proximal plate pierced with four holes for iliac screw fixation and a distal hook designed to engage under the acetabular teardrop or the inferior margin of the obturator foramen, and a horizontal component formed by two lateral flanges [12, 13]. This cruciform geometry gives the device its common name, the “Kerboull cross-plate”, and provides several points of anchorage to host bone, i.e.; the iliac plate, the inferior hook, and, when residual acetabular walls remain, the horizontal flanges, while leaving open windows between the branches through which the underlying bone graft can be directly loaded and can progressively incorporate [12, 13].

**Fig. 3:**
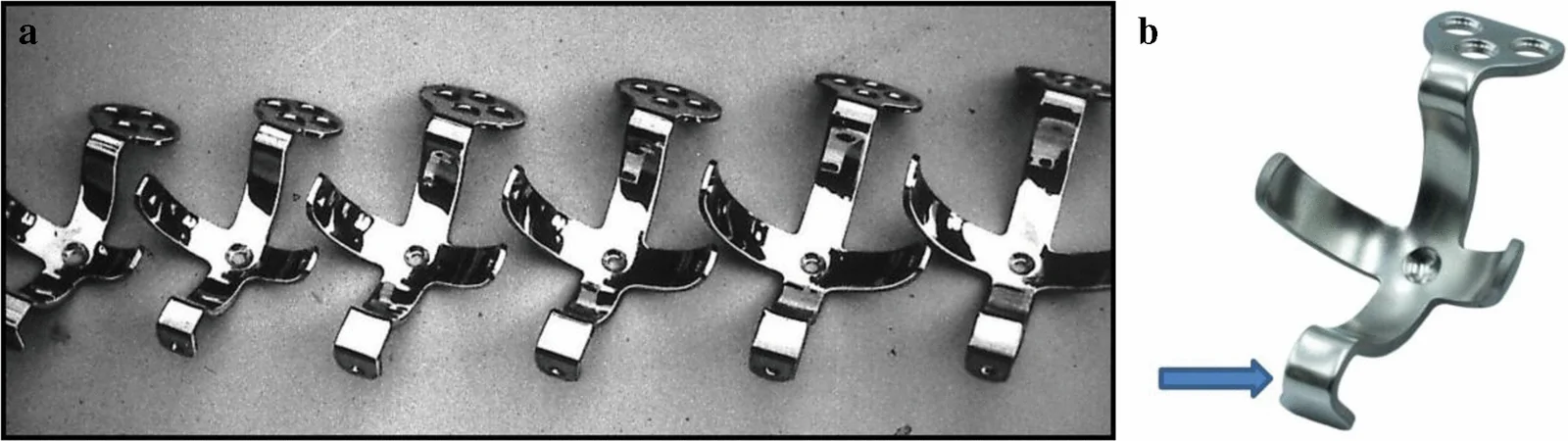
Original and modified Kerboull cross-plate. **A** The original device, manufactured in 316L stainless steel and available in six sizes (Biomet, Warsaw, IN, USA). **B** The modified Kerboull cage, manufactured in Grade 4 surgical titanium, with an enlarged hook (arrow) (courtesy of Medacta SA, Castel San Pietro, Switzerland)

Two features distinguish the design philosophy of the Kerboull cross-plate from other reinforcement constructs of the same era. First, rather than simply bridging a defect on the available residual host bone, the device is positioned so as to reproduce the anatomical centre of rotation of the hip, using the hook and the plate as anatomical landmarks; this deliberate emphasis on restoring acetabular bone stock and hip biomechanics, rather than only providing a stable scaffold, is a recurrent theme throughout the subsequent literature describing the device [4, 10, 12, 13]. Second, the device functions as a temporary, load-sharing internal splint: it is intended to protect the graft during its incorporation phase, but is not conceived to provide definitive, isolated long-term fixation independent of biological healing of the graft [4, 14, 15]. Adaptations of the original design, marketed under various trade names as “Kerboull-type” (KT) plates, have subsequently been developed, particularly in Japan, with variations in dome diameter, hook length and material, while preserving the original cruciform concept [16]. More recently, the device has been manufactured in Grade 4 titanium (ASTM F67) instead of stainless steel, as titanium alloys generally exhibit greater fatigue resistance than 316L stainless steel [17]. The 2.5-mm plate thickness was maintained to preserve construct rigidity [17]. The hook has also been enlarged to address cases with partial alteration of the inferior acetabular margin, and the outer surface sand-blasted to enhance primary stability. The device (Kerboull cage®, Medacta, Castel San Pietro, Switzerland) is available in five sizes with dedicated ancillary instrumentation, and accepts cemented conventional full-polyethylene or dual mobility acetabular components ranging from 40 to 60 mm (Fig. 3).

## Biomechanical rationale

The mechanical rationale of the Kerboull cross-plate, shared with other acetabular reinforcement devices, is to act as a load-sharing splint that unloads the underlying bone graft during the period required for its biological incorporation, thereby limiting the risk of early graft collapse and resorption, and subsequent implant migration. This rationale has been directly quantified using finite-element analysis. Kawanabe et al. compared, in a simulation model of RTHA, the load-dispersion properties of several acetabular reinforcement devices, including the Kerboull cross-plate, the Ganz ring, the Müller ring and the Burch–Schneider antiprotrusio cage, loaded through a graft-cup construct under a simulated single-leg-stance hip contact force [5]. Across the reinforcement constructs modelled, the stresses transmitted to the underlying bone graft were substantially lower than those generated by a cemented cup implanted directly onto the acetabular bone without any reinforcement device, consistent with a genuine protective, load-sharing effect of the reinforcement plate on the graft during the incorporation period [5]. However, this study further suggested that, in contrast to the three other reinforcement devices modelled, high-stress areas with the Kerboull cross-plate were localised in the hook and the bend of the plate rather than on its inner surface, thereby diverting mechanical stress away from the load-bearing dome of the cemented acetabular component and from the structural and morselised allograft, therefore protecting the allograft from high stress during osseointegration, limiting the risk of collapse [5, 6].

This protective effect was corroborated clinically by Akiyama et al., who combined a minimum 4.5-year follow-up study of a Kerboull-type device used with bone allograft with a complementary mechanical analysis of the construct, and reported an absence of mechanical failure of the plate together with satisfactory allograft incorporation in the large majority of hips [14]. Taken together, the finite-element and clinical-mechanical data support the concept that the cruciform geometry of the Kerboull cross-plate, by distributing the hip joint reaction force across the iliac plate, the inferior hook and the horizontal flanges, reduces the mechanical demand placed on the graft compared with an unprotected cemented cup, an effect that is expected to be most relevant for large structural grafts, which are inherently more vulnerable to early mechanical overload than well-vascularised host bone [5, 14].

## Surgical technique

The surgical technique described by Kerboull and Hamadouche relies on a wide surgical exposure of the acetabulum, most commonly through a lateral approach with trochanteric osteotomy or a postero-lateral approach, allowing complete removal of the failed component and of any residual cement, followed by thorough debridement of the fibrous membrane and granulation tissue lining the defect and by pulsatile lavage of the acetabular cavity [4, 12]. Aside from debridement down to bleeding host bone, best performed with curettes. Reaming of the acetabular cavity is deliberately avoided, since the residual acetabular walls are typically thin as a consequence of the pre-existing bone loss, and further bone removal would only aggravate the defect [12].

**Fig. 4:**
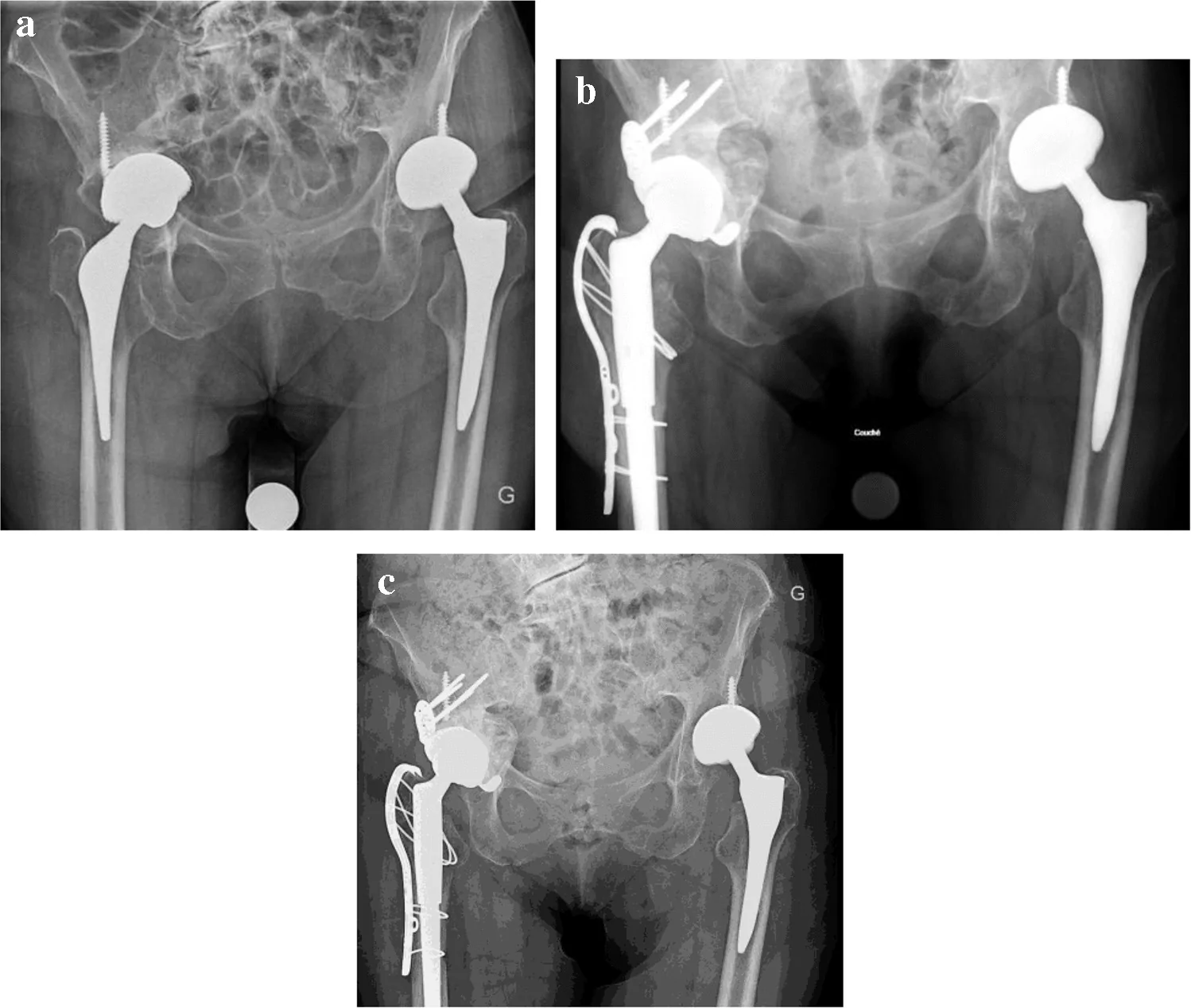
Representative case of Kerboull cross-plate reconstruction with structural and morselised allograft and a cemented dual mobility cup during both components revision total hip arthroplasty associated with Paprosky IIC acetabular bone defect in a 76-year-old woman. **A** Pre-operative, **B** 1-year post-operative and (**C**) 5-year post-operative radiographs demonstrating restoration of anatomical location of hip centre of rotation and complete osteointegration of the allograft

**Fig. 5:**
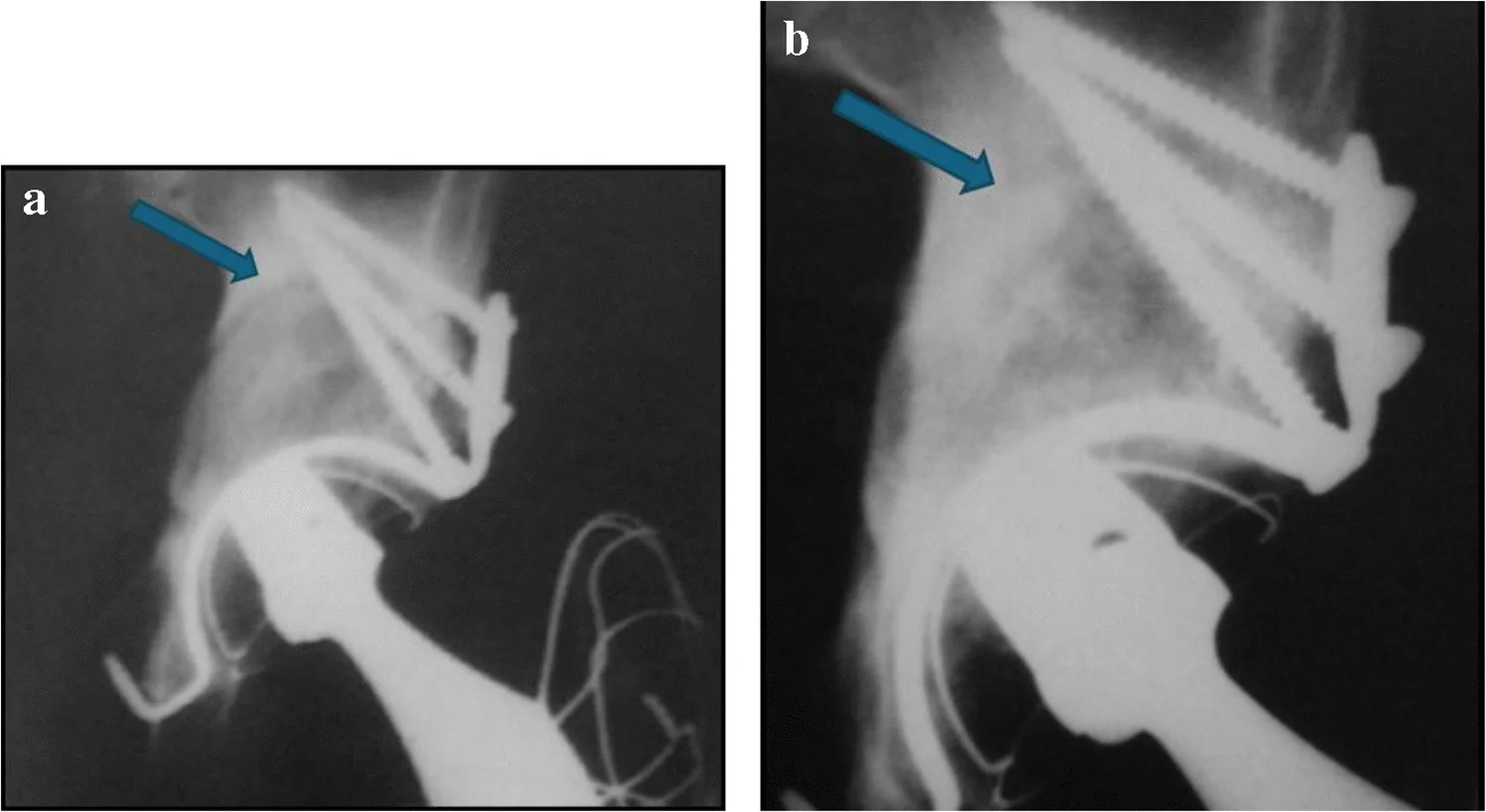
Major acetabular reconstruction (Paprosky IIIA acetabular bone defect) performed with structural bone allograft and a Kerboull cross-plate. **A** Immediate postoperative radiograph; the arrow indicates the demarcation line between host bone and allograft bone. **B** Radiograph at 12-month follow-up showing osseointegration of the graft with disappearance of the demarcation line between host bone and allograft bone; allograft bone remodelling can also be seen

**Fig. 6:**
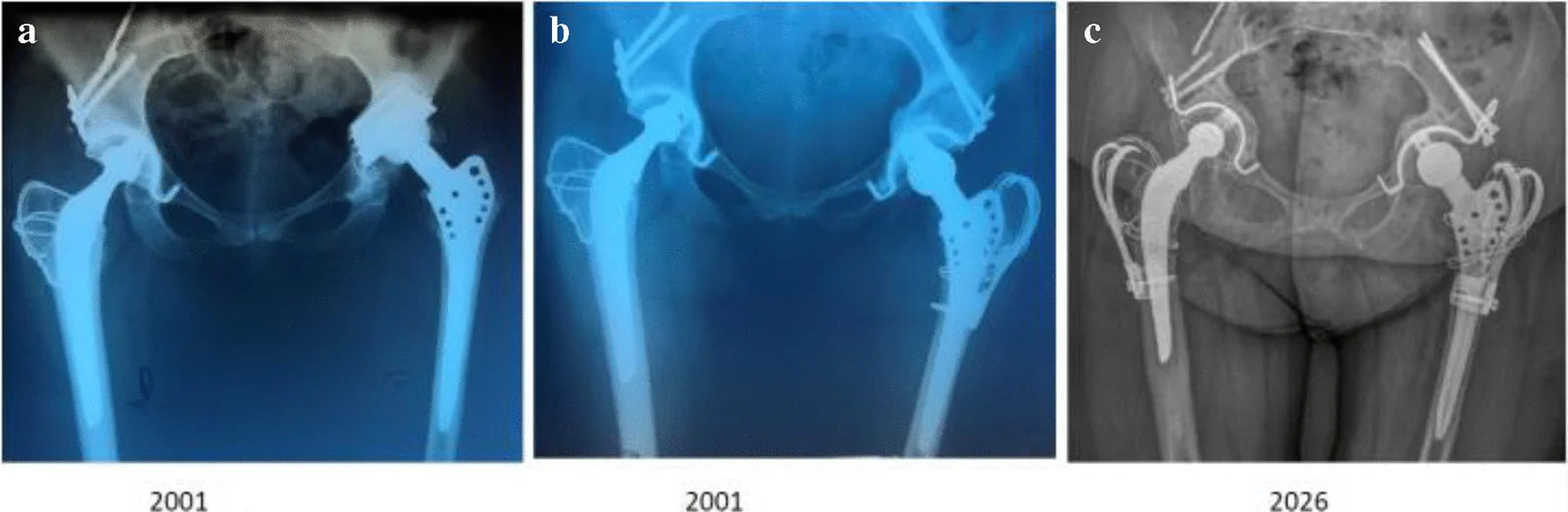
Long-term outcome of a Kerboull cross-plate reconstruction using a contralateral femoral head autograft in a 35-year-old woman with rheumatoid arthritis. **A** Antero-posterior pelvic radiograph showing bilateral primary total hip arthroplasty, cementless on the left side, at 6-month (right) and 7-year (left) follow-up; the left acetabular component shows wear, osteolysis and proximal migration, while the femoral components remain well fixed on both sides. During right-side primary total hip arthroplasty, the femoral head was cryopreserved as an autograft for the subsequent left-side revision. **B** Immediate postoperative radiograph after left-side cup revision using a Kerboull cross-plate reconstructed with the cryopreserved contralateral femoral head autograft. **C** At 25-year follow-up, the left side shows no wear and excellent function, whereas the unrevised right side shows wear with limited migration

**Fig. 7:**
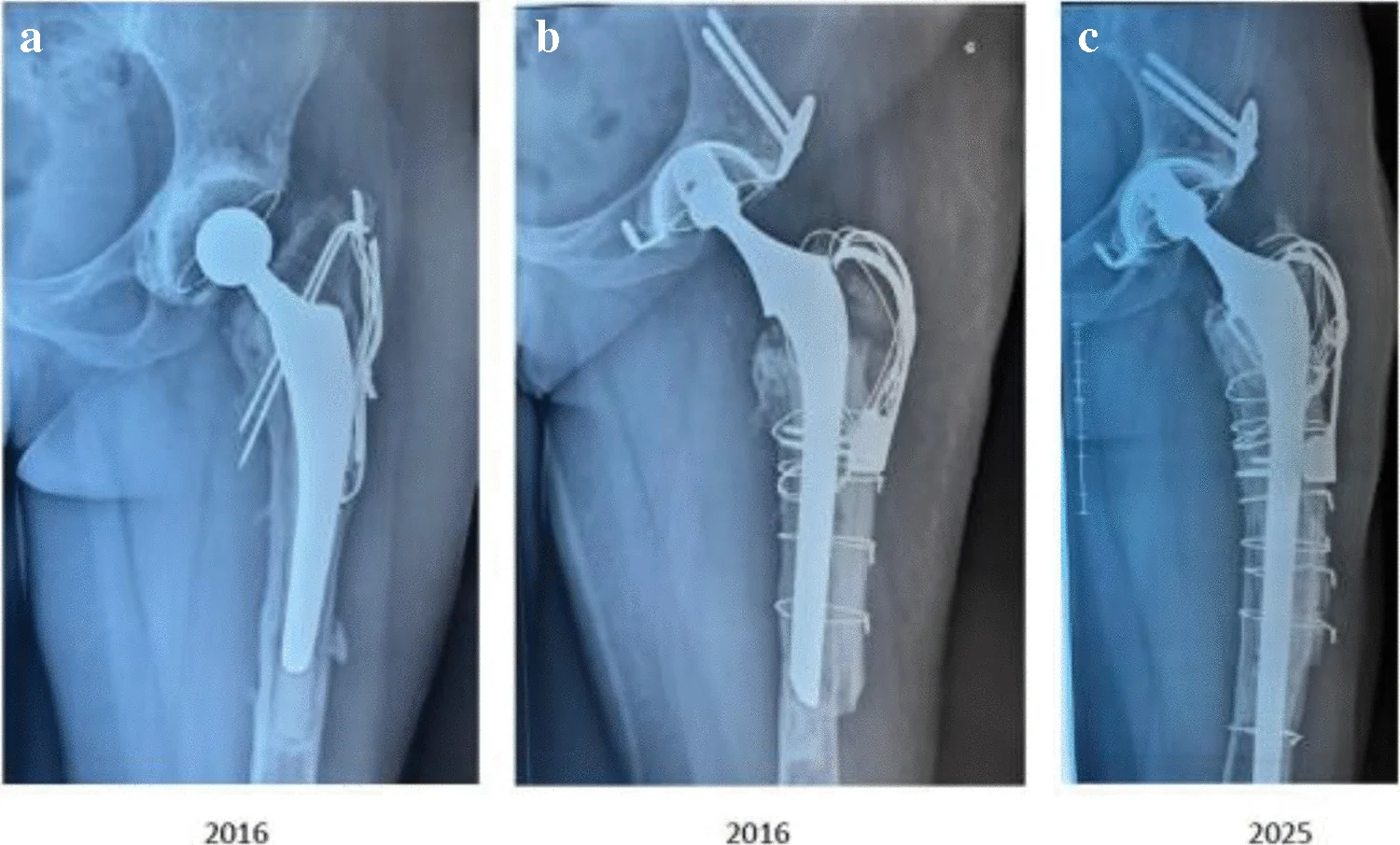
Kerboull cross-plate reconstruction in a technically demanding revision setting. **A** Preoperative radiograph of the left hip of a 45-year-old woman after multiple prior revision total hip arthroplasties, including a two-stage revision for periprosthetic infection; this radiograph, obtained 4 years after the fourth revision, shows periprosthetic osteolysis on both the femoral and acetabular sides, with negative preoperative aspiration. **B** Postoperative radiograph showing combined acetabular and femoral revision using allograft bone and a cemented Kerboull cross-plate reconstruction. **C** At 9-year follow-up, the acetabular reconstruction shows favourable evolution; the femoral component was revised at 4 years for insufficient grafting

Once the cavity has been prepared, the inferior hook of the Kerboull cross-plate is engaged under the acetabular teardrop, or under the inferior margin of the obturator foramen when the teardrop itself has been resorbed, before the plate is applied against the iliac bone at a 40° to 45° abduction angle (horizontal to the floor) with neutral anteversion and provisionally held in position [4, 12, 16]. Positioned in this way, the Kerboull cross-plate defines the size and shape of the allograft required for acetabular bone stock reconstruction, according to the remaining bone landmarks identified after debridement, and automatically restores an anatomical acetabular size, location and hip centre of rotation. The remaining segmental and cavitary defects are then filled with bone allograft, i.e.; structural where mechanical support is required for a segmental defect, morselised for residual cavitary defects, before the plate is fixed to the iliac bone with two to four bi-cortical 4.5-mm or 5-mm stainless-steel or titanium screws according to the cast material of the Kerboull cross-plate [4, 12, 18]. The final reconstruction should provide a contained acetabular cavity with sufficient structural support for the reinforcement device. The four screws are directed at 45° to the horizontal and 10° posteriorly, towards the supra-acetabular and sacroiliac region, taking care to avoid penetration of the sacroiliac joint. The most distal screw is placed first, so as to tension the plate, which must be immediately and perfectly stable on the reconstructed bony acetabulum once compressive screwing is complete. The acetabular component (a cemented conventional full-polyethylene or a dual mobility cup, according to the perceived risk of postoperative instability) is then sized, oriented, and cemented within the Kerboull cross-plate to achieve an adequate and homogeneous circumferential cement mantle [4, 6, 10, 19] (Fig. 4, 5, 6, 7).

A comparable emphasis on anatomical, landmark-based positioning of the Kerboull cross-plate has also been described for the direct anterior approach, in which the teardrop lies in the antero-inferior part of the exposed acetabulum with the patient supine, and its identification may be assisted by intraoperative fluoroscopy [16]. Finally, cementation of the acetabular component is performed once the graft has been secured beneath the plate, respecting an adequate cement mantle thickness and the planned inclination and anteversion of the component [4, 12, 13].

**Fig. 8:**
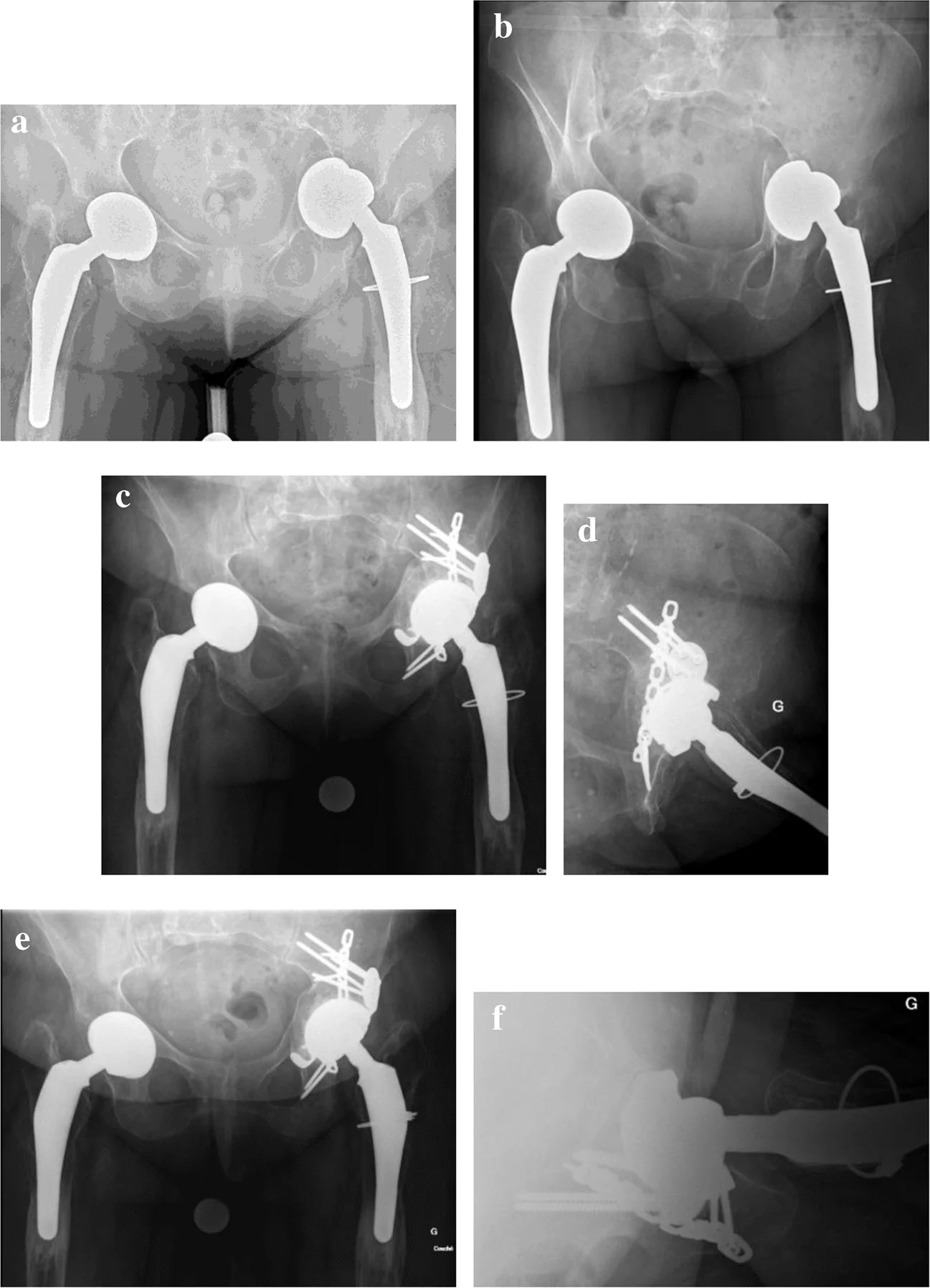
Representative case of post-traumatic acetabular loosening with pelvic disconitunuity combining posterior column reconstruction and a Kerboull cross-plate reconstruction with cemented dual mobility cup in a 64 year-old man. **A**–**F** Pre-operative and sequential follow-up radiographs, including detail views of the reconstruction

A relative contraindication to the Kerboull cross-plate is pelvic discontinuity, whether due to wear-induced osteolysis or to an acetabular periprosthetic fracture. In these situations, stabilisation of the posterior column is a prerequisite, requiring reduction and internal fixation with a reconstruction plate before acetabular reconstruction according to the Kerboull technique, or the use of a Burch–Schneider antiprotrusio cage (Fig. 8).

The ten technical principles that, in our experience, govern a reproducible Kerboull cross-plate reconstruction are summarised in Table 1.

**Table 1:** Ten technical rules for Kerboull cross-plate reconstruction

Rule	Principle	Description
1	Find the true acetabulum	Do not allow the failed implant or the enlarged revision cavity to determine the new hip centre
2	Preserve host bone	Remove non-viable tissue and failed material but avoid unnecessary reaming of viable acetabular bone
3	Identify the teardrop	The inferior acetabular margin (teardrop) provides the key landmark for positioning the hook
4	Reconstruct the acetabular bone stock	The Kerboull cross-plate defines the size and shape of the allograft required for bone stock reconstruction according to the remaining bone landmarks. Cavitary defects are filled with morselised cancellous allograft; segmental defects require structural reconstruction
5	Reconstruct the weight-bearing dome	Structural allograft should restore the deficient load-bearing acetabular surface rather than simply fill the defect
6	Restore the hip centre	The plate should be sized and positioned to restore the anatomical hip centre
7	Engage the hook securely	The hook should sit beneath the teardrop and provide stable inferior support
8	Achieve reliable iliac fixation	The plate should be firmly applied and secured with appropriately directed bi-cortical screws
9	Create a continuous cement mantle	The cemented acetabular component must be secured within the Kerboull cross-plate
10	Use a dual mobility cup for a reason	A dual mobility cup should be selected according to the patient’s instability risk, not simply because the defect is severe

## Principles of bone grafting

### Structural versus morselised graft

A recurring principle in the literature about the Kerboull cross-plate, and on acetabular reinforcement devices in general, is that the nature of the graft used should be dictated by the type of bone defect rather than by surgeon’s habit or availability alone. Segmental structural defects, in which a portion of the acetabular wall or dome is entirely absent and mechanical support must be re-created, require a structural graft (most commonly a bulk femoral head allograft) shaped to restore the missing bony architecture and to provide immediate mechanical continuity for the device and, subsequently, for the acetabular reconstruction. Contained cavitary defects, in which the acetabular walls remain in continuity but bone stock is deficient, are, by contrast, appropriately managed with morselised graft, which role is limited to filling the cavity and providing an osteoconductive substrate for bone stock restoration, without being expected to bear structural load on its own.

This distinction is supported by comparative clinical series. Kawanabe et al. reported the outcomes of 42 hips reconstructed with the Kerboull cross-plate, in which morselised autogenous or ceramic-particle graft had been used even for large defects in the earlier part of the series, before a change in practice towards bulk femoral head allograft for large defects. The authors reported, at ten year follow-up, a survival free of radiological failure was 53% in the morselised-graft group, compared with 82% in the bulk-graft group. The authors concluded that outcomes were better with bulk graft irrespective of defect size [15]. Similarly, Makita et al., comparing bulk and morselised femoral head allograft used with a Kerboull-type plate, reported markedly higher survivorship with bulk graft when the defect required structural support [18], and Hori et al., in a mid-term series in which bulk allograft was used in the large majority of type III and IV defects, reported ten year survival free of re-revision of 92.3% [20]. Taken altogether, these data support the principle that, when a structural defect is present, a structural graft, rather than impaction of morselised bone alone, should be used to obtain a mechanically viable reconstruction. Whereas, morselised graft must be reserved for the completion of contained cavitary filling around the structural graft or the device.

### Graft quality: cryopreserved femoral head versus irradiated allograft

In addition to the choice between structural and morselised graft, the quality of the allograft tissue itself is a major determinant of its incorporation and, ultimately, of its mechanical reliability once loaded beneath the reinforcement device. Bone banks process femoral head allografts using different protocols, ranging from deep-freezing or cryopreservation with minimal further processing, to terminal sterilisation by gamma irradiation, which is used by many tissue banks specifically to further reduce the residual, even though already very low, risk of disease transmission associated with properly screened musculoskeletal allografts.

Terminal irradiation, however, is not without biological and mechanical consequences. In a review of the literature on allograft sterilization process, Nguyen et al. concluded that gamma irradiation adversely affects both the mechanical and the biological properties of bone allografts, through collagen cross-linking and dose-dependent degradation of the collagen matrix, with a clear reduction in mechanical strength once doses exceed approximately 25 kGy (2.5 Mrad) for cortical bone, together with evidence of reduced osteoclastic activity and altered graft remodeling at the biological level [21]. For a large structural bulk graft used beneath a Kerboull cross-plate, in which mechanical continuity and progressive biological incorporation of the graft are both required for long-term success, these adverse effects of irradiation are of direct clinical relevance.

On this basis, when the surgical strategy relies on the protective, load-sharing effect of the Kerboull cross-plate to allow biological incorporation of a large structural graft, as opposed to a purely mechanical filler, cryopreserved femoral head allograft, processed with minimal irradiation, should be favoured over heavily irradiated allograft bone, in order to preserve as much of the graft’s native mechanical strength and osteogenic potential as possible. This should be regarded as a rational, biomechanically and biologically grounded recommendation, supported by laboratory data on the effects of irradiation on allograft bone [21], rather than as a conclusion drawn from a direct, dedicated randomised comparison of cryopreserved versus irradiated allograft specifically used with the Kerboull cross-plate, which, to our knowledge, has not been performed. The latter represents an identified clinical gap that could usefully be addressed by future comparative studies.

## Clinical outcomes

### Outcomes with cemented conventional full-polyethylene acetabular components

The original series reported by Kerboull, Hamadouche and Kerboull established the long-term reliability of the device when combined with a cemented conventional full-polyethylene cup, with a Kaplan–Meier survival rate free of aseptic loosening of 92.1% at 13 years, in 60 hips with AAOS type III or IV defects followed for a mean of 10 ± 3 years [4]. This has since been corroborated, with some nuance, by independent series. Gibon et al., focusing specifically on the technically demanding subgroup of Paprosky type III defects involving destruction of the inferior acetabular margin, reported, in 37 hips reconstructed with the Kerboull device, fresh-frozen femoral head allograft and reconstruction of the inferior margin, a significant improvement in the Merle d’Aubigné score from a mean of 12.5 preoperatively to 16.5 at a mean follow-up of 8.2 years; the authors nevertheless reported acceptable, rather than excellent, mid-term results, with a notable 21.6% rate of radiographic migration of the acetabular component, underlining that reconstruction of the inferior margin remains one of the most demanding indications for the device [22]. This extremely complex situation where the inferior margin of the acetabulum has been obliterated could benefit from the newly design Kerboull® cage (Medacta) that includes a larger hook. In Japan, Baba and Shitoto compared outcomes of the original Kerboull plate and of the modified Kerboull-type plate in RTHA and reported favourable mid-term survivorship with both implants, supporting the durability of the cruciform design across its various iterations [23].

### The rationale for cemented dual mobility acetabular components

Postoperative instability remains one of the most frequent complications of RTHA, and this risk is compounded when extensive acetabular bone loss and abductor deficiency coexist, as is typically the case in patients requiring a Kerboull cross-plate. Independently of any specific association with the Kerboull device, dual mobility cups have been shown to reduce the risk of dislocation in complex revision settings, whether cemented directly into host bone stock or into a metal shell. This has been reported in several series by Hamadouche et al. and Wegrzyn et al. on the use of cemented dual mobility cups to treat or prevent recurrent instability, with sustained benefit at five- to thirteen-year follow-up [8, 24–26].

Building on this rationale, cementation of a dual mobility cup directly within the Kerboull cross-plate has been increasingly reported as a means of combining the structural and load-sharing benefits of the device with the additional stability afforded by a dual mobility bearing. Wegrzyn et al. reported the outcomes of a continuous, prospective series of 61 RTHAs with AAOS grade III or IV acetabular defects reconstructed with a Kerboull cross-plate, structural allograft and a cemented dual mobility cup, and observed no instability at a mean follow-up of 7.5 years, together with complete allograft osseointegration and an absence of mechanical rupture of the plate or loosening of the cemented dual mobility cup in 98% of hips [6]. A further potential indication in demanding and difficult bone condition during total hip arthroplasty was explored by Wegrzyn et al. in patients with periacetabular metastatic disease, combining tumour curettage and cementation of a Kerboull cross-plate with a cemented dual mobility cup; the dislocation rate was 2%, and no mechanical failure or aseptic loosening of the acetabular reconstruction was observed at a mean follow-up of 33 months, a combination the authors considered particularly attractive in patients expected to undergo adjuvant radiotherapy and to survive long enough for durable reconstruction to matter [7].

Assi et al. subsequently reported a direct comparison of cemented conventional full-polyethylene cups and cemented dual mobility cups used within a Kerboull cross-plate reconstruction, and found a markedly lower dislocation rate with the dual mobility bearing, supporting its preferential use whenever the Kerboull cross-plate is indicated in a revision setting at elevated risk of instability [27]. In a larger, longer-term series from the same group, Assi et al. subsequently reported outcomes of a comparable construct used specifically in first-revision THA for major acetabular defects, at a maximum follow-up of fourteen years, again with favourable clinical and radiological results and no recorded dislocation [28].

Taken as a whole, the accumulated experience with cementation of a dual mobility cup within the Kerboull cross-plate suggests that this combination reproduces the long-term structural reliability of the original construct while substantially mitigating its historical Achilles’ heel, namely postoperative dislocation, and this construct can reasonably be considered the current reference option whenever a Kerboull cross-plate reconstruction is undertaken in a patient at high risk of instability [6, 27, 28]. However, to the best of the authors’ knowledge, long-term outcomes of major acetabular reconstruction using this combination have not yet been reported. Although dual mobility cups probably partially buffer the stress transmitted across the cemented interface, the Kerboull cross-plate, and the underlying bone [29], cementing a metal-shell within the device remains a potential concern given the marked differences in Young’s modulus between the three materials.

## Current indications

On the basis of the evidence summarised above, the Kerboull cross-plate should currently be considered for major, combined segmental and cavitary acetabular defects (typically AAOS type III and IV, or Paprosky type IIB to IIIA defects) in which residual host bone cannot provide adequate primary coverage for a cementless component, and in which restoration of the anatomical centre of rotation and of acetabular bone stock through structural grafting is a therapeutic objective, rather than a purely salvage, non-anatomical solution [4, 5, 18, 22].

Cementation of a dual mobility cup within the device should be favoured over a conventional full-polyethylene cemented cup whenever risk factors for postoperative instability are present, which is frequently the case in this population, i.e.; multiple prior surgeries, abductor deficiency, or a documented history of dislocation, given the consistent reduction in dislocation risk reported with this combination without apparent compromise of the mechanical reliability of the reconstruction [6, 27, 28]. Conversely, in the presence of severe pelvic discontinuity or of massive, non-reconstructible bone loss, alternative solutions such as cup-cage constructs, custom-made triflange components, or, in the tumour setting, dedicated pelvic reconstruction techniques, remain more appropriate, and the Kerboull cross-plate should not be regarded as a universal solution for every degree of acetabular bone loss.

## Is the Kerboull cross-plate still relevant in the era of porous metal?

The emergence of porous metal augments and custom-made triflange components has not made the Kerboull principle obsolete; rather, it has clarified its appropriate role. Porous metal constructs are particularly attractive when biological fixation to viable host bone can be obtained and when immediate structural stability is required. The Kerboull construct, conversely, remains highly attractive when a surgeon wishes to reconstruct deficient bone stock with allograft while using a cemented acetabular construct. The key distinction is therefore philosophical: a highly porous revision cup attempts to achieve fixation despite bone loss, whereas the Kerboull strategy attempts to reconstruct bone loss and to protect the reconstruction during biological incorporation.

Neither philosophy is universally superior; the two address different anatomical situations. In the elderly patient with severe bone loss, limited biological potential and a high risk of instability, the combination of a reinforcement device, allograft and a cemented dual mobility cup remains particularly rational. In contrast, a younger patient with sufficient host bone for biological fixation may benefit from a porous metal construct designed for long-term osseointegration.

## Conclusion

From its original description at Cochin University Hospital to its current use with a cemented dual mobility cup, the Kerboull cross-plate has remained a mechanically viable and biologically rational solution for the reconstruction of major acetabular bone loss in RTHA. Its cruciform design provides a genuine, finite-element-validated load-sharing effect that protects the underlying bone graft, provided that the surgical technique respects the anatomical principles originally described, that a structural graft is used whenever a structural defect must be reconstructed, and that the biological quality of the allograft (cryopreserved rather than heavily irradiated) is preserved. The addition of a cemented dual mobility cup within the device now allows this historically reliable construct to also address the risk of postoperative instability that has long represented its main clinical limitation, and current evidence supports this combination as the reference option for the most demanding acetabular reconstructions encountered in RTHA.

## Data Availability

No datasets were generated or analysed during the current study.
